# Influence of age and sex on platelet count: Implications for optimizing growth factor-rich plasma preparation

**DOI:** 10.4317/jced.63137

**Published:** 2025-10-17

**Authors:** Arturo Sánchez-Pérez, Ana Palma-Sánchez, Alfonso Jornet-García, María José Moya-Villaescusa, José María Montoya-Carralero

**Affiliations:** 1 Department of Periodontology. Faculty of Medicine and Dentistry. University of Murcia. Spain; 2 Dental Graduate. University of Murcia

## Abstract

**Background:**

Growth factor-rich plasma (GFRP) is a biomedical procedure used to promote tissue regeneration. Platelet-rich plasma (PRP) contains higher-than-average platelet concentrations and includes platelet-derived growth factors. As an autologous blood derivative, it is widely used to enhance healing and tissue regeneration . In dentistry, GFRP accelerates soft tissue healing and bone regeneration in procedures such as complex extractions, bone grafts, cyst treatments, and to improve dental implant osseointegration. Objective: To assess the potential influence of age and sex on platelet count, with the aim of optimizing the standardization of therapies involving growth factors.

**Material and Methods:**

A cross-sectional, observational, and comparative study was conducted, including a total of 384 patients. Participants were stratified into six groups based on age. From their medical records, the platelet counts obtained from analyses meeting the established inclusion criteria were recorded. Subsequently, the distribution of platelet levels was analyzed according to age groups and sex. Additionally, the potential correlation between platelet count and age was evaluated.

**Results:**

The mean platelet count was higher in women (260.9 ± 65.9 x10³/µL) than in men (250.4 ± 62.0 x10³/µL), although the difference was not statistically significant (p = 0.113). A statistically significant negative correlation was found between age and platelet count (r = -0.175; p &lt; 0.01), indicating that platelet count decreases with age, although the correlation coefficient was low.

**Conclusions:**

Platelet count varies across the population; therefore, blood extraction for PRP preparation should be adjusted based on age group and sex.

## Introduction

Growth factor-rich plasma (GFRP) is a regenerative biomedical therapy that promotes tissue repair. Platelet-rich plasma (PRP) is a plasma fraction obtained by blood centrifugation, producing a platelet concentrate above baseline levels and enriched with associated growth factors. This autologous blood product contains elevated concentrations of bioactive proteins, including platelet-derived extracellular vesicles, which may enhance regeneration or repair of specific tissues and organs (1 , 2). Some studies suggest that for therapeutic efficacy, platelet concentrations should range between two to six times above baseline values (3). A further development of PRP is platelet-rich fibrin (PRF), which allows for sustained release of growth factors and enhances tissue No dropouts were expected, as the study was based on patient medical records regeneration via its natural fibrin matrix. Unlike PRP, PRF is processed without additives, allowing it to be used directly after centrifugation or in membrane form following compression. The regenerative effects of PRP and PRF are attributed to the presence of biological factors and proteins such as fibronectin, 1-antitrypsin, chemotactic factors, and acid phosphatase. Key growth factors derived from plasma and platelets include platelet-derived growth factor (PDGF), transforming growth factor-beta (TGF-), epidermal growth factor (EGF), insulin-like growth factor (IGF), and vascular endothelial growth factor (VEGF), which are locally released upon application (4). The DEPA classification (Dose of injected platelets, Efficiency of production, Purity of PRP, and PRP Activation) is used to categorize PRP quality, with the "AAA" score representing the highest standard and "DDDB" the lowest (5). In oral surgery, PRP is used to support soft tissue healing and bone regeneration in procedures such as complex tooth extractions, bone grafting, or cyst management (6). In periodontics, it has shown favorable results in periodontal tissue regeneration for osseous defects, with techniques including flap debridement or tissue grafting (7). In implantology, PRP has demonstrated efficacy in enhancing dental implant osseointegration and accelerating peri-implant bone formation (8). Given that the therapeutic efficacy of PDGF depends on platelet quantity and quality, it is essential to establish a relationship between platelet count and growth factor concentration. Additionally, platelet count may be influenced by factors such as age and sex, potentially affecting PDGF yield and treatment outcomes. We hypothesized that platelet count declines with age and differs by sex, potentially affecting GFRP efficacy. Therefore, the objectives of this study are to determine whether significant differences in platelet count exist based on age, to assess if sex significantly influences platelet count, and to evaluate the combined impact of age and sex on platelet count to optimize GFRP collection and preparation.

## Material and Methods

**Figure 1:**
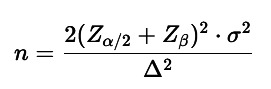
Formula.

This was a cross-sectional, observational, and comparative study conducted at the José Luis Morales Meseguer General University Hospital (HMM) in the Region of Murcia. The study received prior evaluation and approval from the Ethics Committee for Health Research of Area VI (Murcia - Vega Media del Segura) on March 26, 2025, under the internal code EST-09/25, in accordance with the ethical principles for medical research involving human subjects set forth in the Declaration of Helsinki (World Medical Association, 1964; latest revision: october 2024). This research did not receive any specific grants from funding agencies in the public, commercial, or not-for-profit sectors and the authors declare no conflict of interest, financial or otherwise. Inclusion criteria: The sample consisted of healthy patients over 18 years of age who met the following criteria: Absence of chronic diseases classified as ASA I or II by the American Society of Anesthesiologists. No active prescriptions for medications used to treat acute conditions, such as: o First-line analgesics, excluding NSAIDs or corticosteroids unless taken more than one month prior to the blood test. o Antibiotics that do not induce thrombocytopenia. o Ophthalmic or otic eye drops that do not affect platelet count. o Inhalers with no impact on platelet function. o Topical medications (ointments, gels, or patches), provided they do not cause thrombocytopenia or affect platelet function. History of acute diseases that resolved without sequelae. Absence of polypharmacy (defined as the use of five or more drugs for chronic conditions) unless these drugs do not interfere with coagulation. No history of splenectomy. Exclusion criteria: History of hematologic disorders or diseases. Active oncologic processes under chemotherapy, immunotherapy, or monoclonal antibody treatment. Use of medications affecting blood coagulation, such as antiplatelet agents, oral anticoagulants (including novel oral anticoagulants), or heparins. Endocrine or autoimmune disorders, including uncontrolled diabetes mellitus (HbA1c &gt; 6.5%) or flare-up diseases requiring medication adjustments that could alter coagulation. Blood tests requested due to suspicion of acute pathology, such as emergencies involving internal or external bleeding. Sample size calculation: Using a 95% confidence level (Z = 1.96), an estimated proportion of p = 0.5, and an absolute margin of error of 0.05, the minimum required sample size was calculated as 384 participants. Assuming a platelet count standard deviation of ~64 ×10³/µL in platelet count, this sample size provided over 90% statistical power to detect differences of at least 15 ×10³/µL in platelet count between comparison groups, with a type I error rate () of 5%. We will use the simplified formula to compare two means (t-test): (Fig. 1).

1

Where: n: sample size per group in this case: Men = 170, Women = 214 (pooled standard deviation): 64.4 (clinically relevant difference): we will test with 15,000 platelets/µL Z/2 = 1.96 95% confidence level ( = 0.05) Z to be calculated for statistical power The 384 patients were divided into six age groups, each comprising 64 participants. No dropouts occurred since data were retrospectively collected from medical records. The participants were grouped as follows: Group 1: 18-29 years Group 2: 30-39 years Group 3: 40-49 years Group 4: 50-59 years Group 5: 60-69 years Group 6: 70 years and older Data were retrieved from the clinical records (CR) using the "Selene SP18" system of the Murcia Health Service. To comply with ethical and data protection standards, strict anonymization procedures were followed. Each patient was assigned a unique code that replaced any personally identifiable information. The following four variables were collected: patient code, age, sex, and platelet count (×10³/µL). Data confidentiality was ensured through a secure information management protocol compliant with data protection legislation. Statistical analysis was performed using IBM SPSS Statistics for Windows, version 19.0 (IBM Corp., Armonk, NY, USA). The analysis focused on descriptive statistics for platelet distribution by age and sex, correlation between age and platelet count, comparison of platelet counts between sexes, and a multivariate model to assess the combined effect of age and sex on platelet count.

## Results

Regarding the descriptive statistical analysis of the sample, the mean, median, and standard deviation of the platelet count were calculated by age group and sex, yielding the following results (Table 1).

Table 1

To evaluate the correlation between platelet, count and age, the Kolmogorov-Smirnov test was used to assess normality. The result was non-significant (p = 0.342), indicating a normal distribution of the sample (Table 2).

Table 2

To determine the correlation between patient age and platelet count, a Pearson correlation coefficient was calculated, considering platelet count as the dependent variable and age as the independent variable Pearson correlation analysis revealed a weak but significant inverse relationship (r = -0.175, p &lt; 0.01). , indicating a statistically significant negative correlation. This result was confirmed using Spearman's rank correlation coefficient, which also showed an inverse and statistically significant correlation (r = -0.174; p = 0.001). These findings were further supported by simple linear regression analysis, which demonstrated highly significant results for age. A progressive decline in mean platelet values was observed starting from the age of 40 (Table 3).

Table 3

To analyze differences in platelet, count between sexes, the Student's t-test was applied. Although the mean platelet count was slightly higher in women (260.9 ± 65.9 ×10³/L) than in men (250.4 ± 62.0 ×10³/L), the difference was not statistically significant (p = 0.11). The combined influence of age and sex on platelet count was examined using multiple linear regression, with platelet count as the dependent variable and age and sex as independent variables. Age remained statistically significant (p = 0.001), whereas sex did not (p = 0.89). Finally, an ANOVA was conducted to assess the relationship between age and sex with respect to platelet count. The results showed a statistically significant effect for age but not for sex (Table 4).

Table 4

## Discussion

Regarding the relationship between sex and platelet count, the mean platelet count was slightly higher in women than in men, although this difference was not statistically significant. Consistent with prior research, our results suggest a trend toward higher platelet counts in women, possibly due to hormonal influences (9 , 10). Similarly, a 2023 study by Christakoudi et al. (11), which analyzed sex-based differences in platelet counts using data from the UK Biobank, found that women exhibited significantly higher platelet counts than men. Notably, this difference persisted even after adjusting for variables such as body size and composition, suggesting a substantial biological basis for sex-related differences in platelet count. Beyond quantitative differences, qualitative variations in platelet function have also been described. From a pathophysiological standpoint, women demonstrate increased platelet reactivity and functionality. Prior studies have shown that female platelets respond more robustly to agonists like adenosine diphosphate (ADP), implying sex-mediated mechanisms in platelet activation (12). These findings underscore the clinical relevance of sex-based differences may have clinical implications, particularly in interpreting biomarkers and tailoring therapies that involve platelet-based treatments. For example, Zhang et al. (2024) observed that higher platelet counts in adult women were associated with lower bone mineral density-an association not observed in men-underscoring the complexity of sex-specific biological interactions (13). With respect to age, our results demonstrated a progressive decrease in platelet count with increasing age. This negative trend was evident in the descriptive analysis by age group, and confirmed through correlation and regression analyses. Although the correlation strength was modest, the association was statistically significant. These findings are in line with those of Rossi et al. (2023), who highlighted how both age and baseline platelet count influence PRP composition. Their study noted a significant reduction in platelet concentration per decade of life, potentially affecting both the efficacy and standardization of PRP preparations (14). Gaetano et al. (2023) also reported a decline in platelet counts with aging, attributing this trend to hematopoietic senescence and decreased megakaryocytic activity (15). Similarly, Le Blanc and Lordkipanidzé (16) observed a significant reduction in platelet count in individuals over 60 years of age, potentially linked to nutritional deficiencies and chronic inflammation. Our results are further supported by Kunapaisal et al. (2023), who observed significantly lower platelet counts upon hospital admission in patients over 50 years old with traumatic brain injury-an association also linked to increased in-hospital mortality (17). These studies collectively reinforce the concept that age directly influences platelet levels, regardless of clinical context, with implications for both prognosis and therapeutic planning. Moreover, studies evaluating both age and sex have found that age-related changes in platelet function may be more pronounced in women than in men (18). Biino et al. (2013), in a study of over 40,000 individuals in Italy, found that women had higher platelet counts than men beginning in adolescence, with values declining with age in both sexes (19). A 2023 study by Gasecka et al. (20) explored the influence of genetic factors, age, and sex on platelet levels and circulating extracellular vesicles. Their findings revealed significantly higher platelet counts in women and confirmed that both age and sex have a considerable impact on these parameters. Thus, our findings are consistent with previous research on the relationship between age, sex, and platelet count, supporting the conclusion that these variables should be considered in the interpretation of platelet indices. These biological differences may also explain individual variability in platelet-based therapies. Statistical analysis revealed that age explained only a small proportion of the total variability in platelet count. This remained true even after including sex as an additional predictor, which did not substantially improve the model's predictive power. Despite the adequate sample size, our study has some limitations, including the absence of complementary clinical information and the exclusion of other hematologic markers that could have enriched the analysis. Limitations Our study did not account for potential confounders such as subclinical inflammation, nutritional deficiencies (e.g., iron or vitamin B12), or hormonal variations (e.g., menstrual cycle phases), all of which may influence platelet counts. Additionally, unmeasured variables-including undiagnosed comorbidities, lifestyle factors (e.g., smoking, alcohol use), or unreported medication intake-could further contribute to interindividual variability in platelet levels. Future studies should incorporate these parameters to enhance the reliability of platelet count assessments for GFRP applications. For instance, Chou et al. (2022) demonstrated a positive association between platelet count and arterial stiffness in young and middle-aged adults, suggesting a potential cardiovascular influence on platelet levels (21). Iron, vitamin B12, and folic acid deficiencies may also alter hematopoiesis and platelet production and survival (22 - 24). These variables could affect PRP quality and should be considered in future research. Other unaccounted variables include hormonal fluctuations during the menstrual cycle, which can impact both platelet count and function. Several studies have shown that mean platelet volume varies across different menstrual phases and that sex hormones such as estradiol can modulate platelet function and even induce apoptosis (25 - 27). These hormonal variations may be particularly relevant in women of reproductive age. Nevertheless, the data presented in this study support the existence of general patterns in platelet distribution based on age and sex, which may have implications for interpreting complete blood counts across different population groups.

## Conclusions

We observed a significant age-related decline in platelet count, particularly after age 60. Women had a slightly higher platelet count than men, although this difference was not statistically significant in our study. Platelet count varies across the population; therefore, blood collection for the preparation of growth factor-rich plasma (GFRP) should be adjusted according to age and sex. Among these factors, age had the greatest influence.

## Figures and Tables

**Table 1:** Mean, median, and standard deviation of platelet count by age group and sex.

		Platelet Count			
		N	Mean	Median	Standard Deviation
Sex	Male	170	250.4	248.3	62.0
	Female	214	260.9	258.0	65.9
Age Group	18-29	64	257.6	255.8	59.2
	30-39	64	281.1	276.5	75.0
	40-49	64	267.3	269.5	59.1
	50-59	64	261.0	263.3	58.9
	60-69	64	229.9	224.3	58.1
	≥70	64	241.0	233.7	63.2
	Total	384	256.2	254.3	64.4

1

**Table 2:** Kolmogorov-Smirnov normality test results.

N		Platelet Count
		384
Normal parameters	Mean	256.21
	Standard Deviation	64.36
Most Extreme Differences	Absolute	0.048
	Positive	0.048
	Negative	-0.029
Kolmogorov-Smirnov Z		0.938
Asymptotic Significance (bilateral)		0.342

2

**Table 3:** ANOVA. Simple linear regression with age as the predictor variable and platelet count as the dependent variable.

Model		Sum of Squares	df	Mean Square	F	Sig.
1	Regression	48348.602	1	48348.602	12.007	.001
	Residual	1538,158.6	382	4026.593		
	Total	1,86,507.2	383			

3

**Table 4:** Predictive variables (age and sex) in relation to the dependent variable (platelet count).

Model		Unstandardized Coefficients		Standardized Coefficients	t	Sig.
		B	Std. Error	Beta		
1	(Constant)	270.992	13.831		19.594	.000
	Age	-0.638	0.181	-0.177	-3.517	.000
	Sex	11.092	6.505	0.086	1.705	.089

4

## Data Availability

The datasets used and/or analyzed during the current study are available from the corresponding author.
